# Electronically Reconfigurable Photonic Switches Incorporating Plasmonic Structures and Phase Change Materials

**DOI:** 10.1002/advs.202200383

**Published:** 2022-04-17

**Authors:** Nikolaos Farmakidis, Nathan Youngblood, June Sang Lee, Johannes Feldmann, Alessandro Lodi, Xuan Li, Samarth Aggarwal, Wen Zhou, Lapo Bogani, Wolfram HP Pernice, C David Wright, Harish Bhaskaran

**Affiliations:** ^1^ Department of Materials University of Oxford Parks Road Oxford OX1 3PH UK; ^2^ Present Address: Department of Electrical and Computer Engineering Swanson School of Engineering University of Pittsburgh Pittsburgh PA 15261 USA; ^3^ Institute of Physics University of Munster Munster Germany; ^4^ Departmentof Engineering University of Exeter Exeter EX4 4QF UK

**Keywords:** integrated opto‐electronics, mixed‐mode PCM, phase change photonics

## Abstract

The ever‐increasing demands for data processing and storage will require seamless monolithic co‐integration of electronics and photonics. Phase‐change materials are uniquely suited to fulfill this function due to their dual electro‐optical sensitivity, nonvolatile retention properties, and fast switching dynamics. The extreme size disparity however between CMOS electronics and dielectric photonics inhibits the realization of efficient and compact electrically driven photonic switches, logic and routing elements. Here, the authors achieve an important milestone in harmonizing the two domains by demonstrating an electrically reconfigurable, ultra‐compact and nonvolatile memory that is optically accessible. The platform relies on localized heat, generated within a plasmonic structure; this uniquely allows for both optical and electrical readout signals to be interlocked with the material state of the PCM while still ensuring that the writing operation is electrically decoupled. Importantly, by miniaturization and effective thermal engineering, the authors achieve unprecedented energy efficiency, opening up a path towards low‐energy optoelectronic hardware for neuromorphic and in‐memory computing.

## Graphical abstract

Light is uniquely suited to transport parallelized information and perform high‐speed computations. In this article, the authors have engineered a path for optical signal modulation, data storage and computation driven by electronics. By confining light to a nanoscale volume through plasmonic structures and by employing active phase‐change materials the authors demonstrate ultra‐low energy, non‐volatile switching with electrical and optical readout.

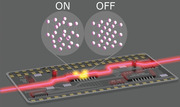

## 1 Introduction

Brain‐inspired computing paradigms are generating unprecedented opportunities in fundamental science and applied technologies. Algorithmic implementations of machine learning are accelerating processing speeds in tasks where humans have traditionally excelled, such as pattern and speech recognition. Yet, the shift toward neuromorphic software has not been accompanied by an equivalent shift in hardware. Computers remain structured based on the von Neumann architecture^[^ ^1^ ^]^ where, unlike biological synapses, the memory and processing units are separated in space and time,^[^ ^2^ ^]^ which results in significant latency and energy penalties, known as the von Neumann bottleneck.^[^ ^3^ ^]^

In overcoming the incompatibility between neuromorphic software and von Neumann hardware, the focus is shifting towards devices that can process and store information simultaneously.^[^ ^2^, ^4^ ^]^ Phase‐change devices have emerged as particularly promising candidates for in‐memory computing as they can switch reversibly between two non‐volatile states with distinct optical^[^ ^5^ ^]^ and electrical^[^ ^6^ ^]^ properties and can thus achieve simultaneously storage and logic functions.^[^ ^7^ ^]^

**Figure 1:**
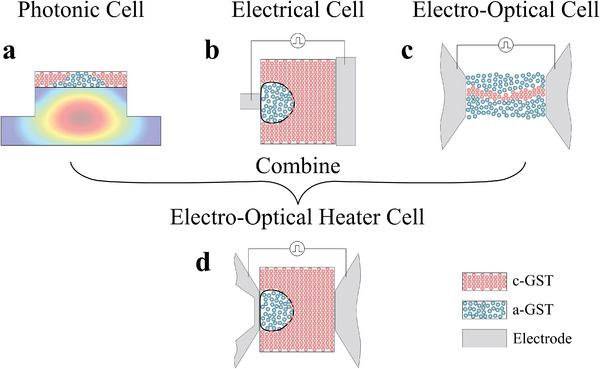
Switching mechanisms in phase‐change devices. a) Integrated photonic phase‐change devices rely on absorption by the PCM to heat the material and induce a phase transition. The temperature decays away from the center of the PCM and coincides with the optical absorption profile. This effect makes up the basis for all‐optical multilevel operation. b) All‐electrical devices rely on direct electrical heating by applying electrical pulses across the PCM. An asymmetric electrode pair induces a thermal gradient across the PCM and results in a hemispherical amorphous volume that can be grown by pulse amplitude modulation enabling multilevel operation. c) Recent demonstration of an electro‐optical cell with symmetric electrodes for simultaneous optical and electrical programming and readout. The electrodes serve to simultaneously route optical and electrical signals. However, this configuration lacks the thermal decay required for multilevel operation. d) Electro‐optical heater cell that combines plasmonic enhancement and indirect heating by a nano‐heater. (this work) The temperature decays away from the heater which enables multilevel operation.

A proliferation of such devices is surfacing rapidly, with phase‐change photonics proving to be capable of high‐speed and bandwidth processing,^[^ ^8^ ^]^ while electrical PCMs demonstrating high scalability and ultra‐low switching energies.^[^ ^9^ ^]^ The prospect of combining the speed of photonics and the efficiency of electronic phase‐change devices has recently attracted significant attention, however, seamless integration of the two domains has remained elusive due to the fundamentally different switching mechanisms involved. This incompatibility between electronic and photonic devices particularly limits the ability to store and retrieve multi‐level information. Achieving high storage density in a single cell is contingent on efficient thermal engineering during SET/RESET operations and depends critically on the programming mechanism deployed. In integrated optical memories, heat is generated by optical absorption in the phase‐change material (**Figure** **1**a). The thermal gradient in the PCM follows the modal profile inducing a direct correlation between the programming power and the state reached.^[^ ^10^ ^]^ This intrinsic property of photonic PMCs has led to an impressive memory density with over 32 distinguishable levels stored in a single cell.^[^ ^11^ ^]^ In electrical PCMs on the other hand, such a thermal gradient does not occur naturally and is forced by thermal engineering at the contacts. An asymmetric electrode pair is designed to induce a higher current density at the interface between the electrode and the phase‐change material.^[^ ^9^, ^12^ ^]^ The heat dissipated creates a near‐hemispherical shape of amorphous material whose size can be tailored by modulating the programming pulses (Figure 1b). Moreover, while optical devices demonstrate superior performance in the readout in terms of speed and bandwidth, their electrical counterparts require substantially lower programming energies.

The realization of electrically programmable but optically addressable phase‐change devices however remains challenging due to the absence of efficient electrical switching mechanisms capable of producing a strong optical response. One approach demonstrated recently by our group has been the implementation of plasmonic structures with electro‐optical functionality. Extreme field enhancement in these structures provides a means of programming the state of a PCM cell via both optical and electrical pulses,^[^ ^13^ ^]^ an approach that has also been successfully employed in free‐space phase‐change photonics.^[^ ^14^ ^]^ While enhanced light–matter interactions in the PCM provided high sensitivity readout and record‐breaking energy metrics for optical switching, electrical programming pulses showed weak coupling to the optical mode, possibly due to the formation of small filament‐like pathways as illustrated in Figure 1c.^[^ ^9^, ^15^ ^]^

An alternative approach is to employ “indirect” electrical switching via the use of embedded on‐chip electrical micro‐heaters, with the phase‐change material being placed on top of the heater. Such an approach decouples the heating of the PCM from its conductance state,^[^ ^16^ ^]^ and has been demonstrated using a variety of heater materials ranging from doped silicon,^[^ ^17^ ^]^ transparent oxides,^[^ ^18^ ^]^ metals,^[^ ^14^ ^]^ and graphene.^[^ ^19^ ^]^ However, these devices have diffraction‐limited footprints, require high operating voltages (and relatively high powers), and are not capable of reading out the state of the device electrically, rendering this configuration ideal for display technologies yet suboptimal for integrated photonics.^[^ ^20^ ^]^

Here, we demonstrate an electro‐optic device based on the general plasmonic approach but which employs indirect switching to program the PCM. In this structure the metallic domains, in addition to guiding optical power, they also function as Joule heating elements that transfer heat to the PCM (Figure 1d). By this approach, the volume of the PCM that is addressed is increased, thereby resolving filamentation limitations, yet maintaining the energy and footprint of the device low.

## 2 Device Outline

**Figure 2:**
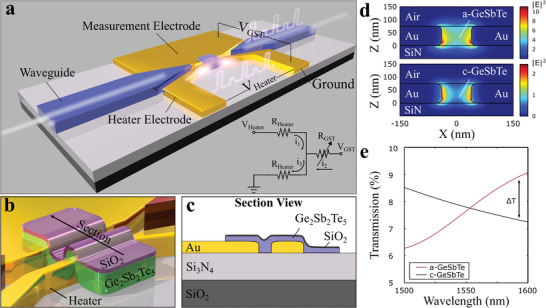
Plasmonic nanoheater device. Light is coupled into a 70 nm wide plasmonic metal–GST–metal waveguide. The material state of the GST across the metal nanogap modulates the transmission state of the device that is programmed by electrical pulses across *V*_Heater_. a) Schematic of the device including Si_3_N_4_ photonic waveguides, the plasmonic MIM waveguide, and the phase‐change element. Two electrical pathways are formed either through the heater (*V*_Heater_) or the GST (V_GST_) shown in the electrical equivalent diagram. Nanosecond pulses across *V*_Heater_ are employed to heat and switch the material while continuous measurement of the state of the GST is achieved by applying a small voltage across *V*_GST_. b,c) Zoomed view and section view of the phase‐change cell consisting of 75 nm Ge_2_Sb_2_Te_5_ and a capping layer of 5 nm SiO_2_ that bridges the nanogap electrodes forming a conductive path between the heater and measurement electrode. d) 2D eigenmode simulations for the case when the nanogap is filled with a‐GST and c‐GST. e) Transmission spectra obtained by 3D FDTD simulations for amorphous and crystalline GST. The device shows higher transmission in the amorphous state at the measurement wavelength.

The device implements a shallow etched Si_3_N_4_ waveguide which is used to efficiently route optical power to a nanoscale gap formed between two gold electrodes as illustrated in **Figure** **2**a. A phase‐change material (Ge_2_Sb_2_Te_5_) occupies the gap between the electrodes and makes up the active region of the device, as shown in the zoom‐in and section views in Figures 2b,c, respectively. Here, the functionality of the electrodes is threefold. First, they form a metal–insulator–metal waveguide (MIM) that is employed to guide and confine light into a nanoscale volume. Eigenmode 2D simulations of a cross section of the device (Lumerical MODE Solutions) are shown in Figure 2d for the case of amorphous (a‐GST) and crystalline (c‐GST). The electric field intensity has been scaled based on 3D finite difference time domain (FDTD) simulations and normalized to the field amplitude of the photonic waveguide, revealing a field enhancement of approximately ten times for the case of a‐GST and a reduction to a twofold enhancement for the case of c‐GST. This intensity enhancement has several advantages, namely lower optical switching energy, higher readout sensitivity, and smaller overall device footprint as previously demonstrated by the authors.^[^ ^13^ ^]^ Upon crystallization, the increase in the refractive index of GST results in an increased field penetration into the metallic domains. The combined effects of field penetration into the metal, absorption by the GST, and differences in the mode coupling between the waveguide and MIM structure in the two material states produces modulated optical transmission levels. Finite difference time domain (3D FDTD) simulations (Lumerical FDTD solutions) show that the amorphous state has higher optical transmission compared to the crystalline state, as shown in Figure 2e.

**Figure 3:**
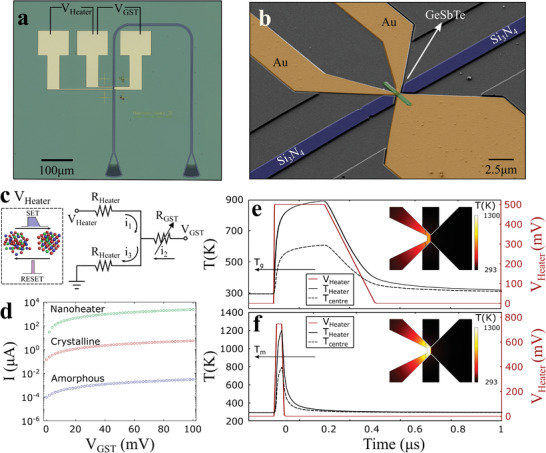
Devices and characterization. Mixed‐mode switching by pulsed operation of the heater (*V*_Heater_) to SET or RESET the device. The device conductance state is measured across the GST (V_GST_). a,b) Optical micrograph and false color SEM of the fabricated device. c) Electrical equivalent circuit of the device. A voltage V_Heater_ is used to switch the state of the GST by localized heating whereas a low bias is applied at V_GST_ to measure the state of the device electrically. d) Demonstration of switching the GST by performing an *I*–*V* scan across *V*_Heater_ (0–600 mV) and measuring the state of the device by conducting an *I*–*V* along V_GST_ (0–100 mV). Crystallization of the device modulates the electrical conductivity by 3 orders of magnitude. e,f) Ohmic heating of the device for crystallization and amorphization respectively.

Second, the electrodes provide a means of measuring the conductance state of the phase‐change cell electrically. A small compliance bias is applied across the nanogap (*V*_GST_ = 100 mV) that is chosen to be well below the threshold for field switching such as to not modify the phase‐state of the device. Third, the electrodes are configured to form a nanoscale constriction of 200 nm width, running along the length of the nanogap as illustrated in Figure 2a. The application of a voltage pulse (*V*_Heater_) across the constriction produces a localized heat source that induces crystallization or amorphization of the phase‐change cell depending on pulse width and amplitude as seen in **Figure** **3**e,f.

## 3 Results and Discussion

The energy required to amorphize is calculated to be ≈650 pJ and that to crystalize the device is found to be ≈4 nJ, which, to our knowledge, are the lowest reported energies for switching phase‐change materials using heaters with integrated optical readout. Our low switching energy is due to the ultra‐compact heating volume in conjunction with the highly sensitive readout enabled by the plasmonic enhancement of the electric field. **Table** **1** summarizes the current state of heater‐based switching of phase‐change materials on integrated photonic platforms, showing that this work reduces the switching energy by more than one order of magnitude for both amorphization and crystallization operations compared to previously reported values.

**Table 1:** Summary of electrical heater‐based switching of PCMs in integrated photonic devices. Only works that demonstrated reversible electrical switching have been summarized above. Table adapted from Ref. [19]

Microheater Material	Amo. Energy [nJ]	Cry. Energy [nJ]	PCM Cell Size [µm^2^]	PCM Material	Mixed‐Mode Readout	Reference
FTO	2100	4400	10 × 10	Ge_20_Te_80_	No	[18a]
ITO	20	2.2 × 10^6^	1.5 × 1.5	GST	No	[18b]
ITO	10	5.1 × 10^6^	1.5 × 2	GST	No	[22]
Metal	5500	4.3 × 10^7^	10 × 10	GSST	No	[5]
Doped‐Si	10	9.0	1 × 1	GST	No	[17a]
Si PIN diode	13	720	0.5 × 5	GST	No	[17b]
	8.0	780	0.5 × 3	GST		
Graphene SiO_2_	290	290 000	3 × 4	GSST	No	[19]
Nanogap heater	0.650	4	0.07 × 0.2	GST	Yes	This work

We use 3D time‐dependent FEM thermal simulations (COMSOL Multiphysics) to evaluate the temperature distribution of the device for amorphization (RESET) and crystallization (SET) pulses. The temperature profiles along with the pulse amplitude are shown in Figure 3e,f, demonstrating the ability of the heater to raise the temperature of the device above the glass transition temperature (*T*_g_ ≈ 430K) and melting temperatures (*T*_m_ ≈ 900 K) of GST, respectively. Here, the values considered are higher than the published average for GST.^[^ ^21^ ^]^ A rectangular 200 ns pulse with a 200 ns linear decay is used to induce crystallization and a 20 ns rectangular pulse with a 5 ns rise and fall time is used to melt‐quench the device to the amorphous state. The simulated transient temperature at the center of the PCM cell (defined as the point located at the midpoint distance between the measurement and heater electrodes and at the half height of the phase‐change cell) can be seen in Figure 3e,f for SET and RESET pulses, respectively. The 2D temperature maps in Figure 3e,f (inset) show the in‐plane temperature distribution at the center slice of the heater where the temperature reaches a maximum and decays along the GST domain. Importantly, the transient temperature profile of the device indicates that the cooling rate is sufficiently fast (*dT*/*dt* > 10^9^ K s^−1^) to melt‐quench the device and induce amorphization.^[^ ^6^ ^]^

Considering the electrical equivalent circuit in Figure 3c including two fixed resistance values at each side of the constriction (*R*_Heater_) that make up the nanoheater as well as the variable resistance of the PCM cell (R_GST_), we perform *I*–*V* measurements across the GST in order to measure the resistance of the devices in their as‐deposited state. One such *I*–*V* measurement is shown in Figure 3d (blue) having a resistance of ≈10 MΩ that is found to scale with the width of the nanogap (Figure S1, Supporting Information). We subsequently perform an *I*–*V* measurement along *V*_Heater_ with a maximum voltage of 600 mV in order to heat the GST and induce crystallization. A final *I*–*V* measurement along V_GST_ reveals that the resistance of the device after crystallization has reduced by 3 orders of magnitude to ≈10 kΩ. The resistance of the device in the crystalline state is shown in Figure 3d (red) as well as the resistance of the heater (green) for reference.

**Figure 4:**
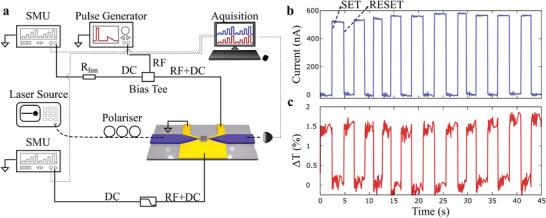
Mixed‐mode operation. a) Optoelectronic measurement setup. The transmission across the device as well as the electrical conductance of the device is programmed by sending electrical set–reset pulses across the heater electrodes. The device is switched through RF pulses across the heater and its conductance state is measured using a source meter unit. A current limiting resistor *R*_lim_ reduces the current through the heater during measurement. b,c) Reversible switching of the device electrically. b) Continuous readout of the current across the PCM. c) Continuous‐wave readout of the optical transmission through the PCM. In accordance with simulated transmission spectra, the device shows higher transmission and lower conductance in the amorphous state.

We demonstrate reversible switching of the device with simultaneous optical end electrical readout by sending electrical pulses through the heater to switch the material. We couple a 5 mW continuous wave (CW) laser at 1585 nm to the device and the optical output of the device was measured continuously using a low noise photodetector (200 kHz bandwidth), as shown in the measurement schematic of **Figure** **4**a. Simultaneously, we measure the current through the PCM cell using a measurement voltage *V*_GST_ = 100 mV across the nanogap. A constant 100 mV bias was applied across the heater electrodes (*V*_Heater_) throughout the experiment in order to detect if any structural changes occurred to the heater by monitoring its resistance, which showed a constant value of 105 Ω. Rectangular SET pulses with an amplitude of 1.2 V, pulse width of 200 ns, and with a subsequent linear decay of 200 ns were sent to the heater in order to crystallize the material, and rectangular RESET pulses with an amplitude of 1.8 V and pulse width of 20 ns to amorphize the device. Figure 4b shows reversible SET‐RESET operation between the low current/high transmission amorphous state and the high current/low transmission crystalline state. Here, we observe an optical contrast, when electrically switching, of 1.5%, a threefold improvement in the contrast of the device compared to previous work^[^ ^13^ ^]^ due to the larger switching volume through the use of indirect electrical switching approach afforded by the nanoheater.

The energy required to amorphize is calculated to be ≈650 pJ and that to crystalize the device is found to be ≈4 nJ, which, to our knowledge, are the lowest reported energies for switching phase‐change materials using heaters with integrated optical readout. Our low switching energy is due to the ultra‐compact heating volume in conjunction with the highly sensitive readout enabled by the plasmonic enhancement of the electric field. Table 1 summarizes the current state of heater‐based switching of phase‐change materials on integrated photonic platforms, showing that this work reduces the switching energy by more than one order of magnitude for both amorphization and crystallization operations compared to previously reported values.

**Figure 5:**
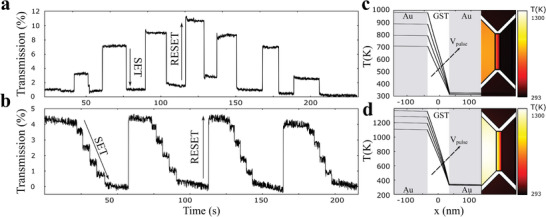
Device programming between multiple states. a) Multilevel operation by amplitude modulation of an amorphization pulse between 2 V and 2.4 V. A single amorphization pulse is applied to program the device to the high transmission state while a single crystallization pulse is applied to return the device to the low transmission state. b) Accumulation property achieved by sending the same crystallization pulse multiple times. c,d) Simulated thermal response with increasing amorphization and crystallization bias.

We further evaluate the ability of the device to program multiple states by modulating the amplitude of the electrical pulses sent to the heater. To demonstrate the ability to reach an intermediate level deterministically from the low‐transmission crystalline state, we send amorphization pulses of varying amplitude separated by a constant crystallization pulse of 1.6 V in order to return the device to the base state. The response from linearly spaced in amplitude amorphization pulses with three increasing and three decreasing pulses from 2 to 2.4 V are shown in **Figure** **5**a. Transmission is modulated by ≈11% that marks an improvement of an order of magnitude over previous work. The mechanism for amplitude modulated multilevel switching is explained by thermal simulations where the pulse amplitude is swept between 400 and 550 mV for crystallization and between 700 and 850 mV for amorphization (Figure 5c‐d). Plotting the temperature as a function of the position along the nanogap, we find that *T*_g_ and *T*_m_ respectively are reached closer to the center of the nanogap for increasing amplitude, making the modulation of the amplitude of the switching pulses capable of controlling the crystalline fractions in the nanogap. We additionally observe an accumulation property of the device wherein the same SET pulse induces further changes in crystallinity (Figure 5b). Five crystallization pulses of 1.5 V in amplitude are sent to the device separated by a 2 V RESET pulse. With every crystallization pulse, a reduction in transmission of 1% is observed while an amorphization pulse returns the device to the amorphous state. We attribute the reduction in transmission caused by identical pulses to further crystallization of polycrystalline domains as well as crystallization of amorphous domains further away from the center of the nanoheater due to the higher thermal conductivity of c‐GST compared to a‐GST.

## 4 Conclusions

We have demonstrated an electrically programmable plasmonic integrated heater coupled to a photonic device. This allows us to reconfigure the phase‐change material Ge_2_Sb_2_Te_5_ electronically, between binary and multilevel states, whilst decoupling the switching from direct injection of current through the material. Our device shows ultra‐low switching voltage operation with pulse amplitudes of 1.4 V to set and 1.8 V to amorphize the device. We prove that in spite of decoupling GST's conductance from the Joule heating mechanism, both optical and electrical readout is not only possible, but results in an increase in the optical readout/modulation contrast by more than an order of magnitude. We measure switching energies down to 4 nJ, the lowest thus far for such devices. These results unambiguously demonstrate the ability to reconfigure optical transmission on a nanoscale platform electrically and being able to access these states optically at low energies and high contrast, opening new avenues in optoelectronic neuromorphic and in‐memory computing that can bridge the gap between the scalability of electronics with the throughput of optics.

## 5 Experimental Section

### Device Fabrication

Standard doped 525 µm Si wafers with a 3.3 µm wet thermal oxide layer and an LPCVD grown stoichiometric Si3N4 layer were obtained from Rogue Valley Microdevices. A first step of electron beam lithography (EBL) was performed on positive tone e‐beam resist (CSAR62) with a charge dissipation layer (ESpacer 300Z) to define the waveguides and grating couplers. After development (AR 600‐546:MIBK:IPA) devices were etched at a depth of 165 nm by dry chemical etching (Oxford Instruments) at 100 W with a gas mixture of CHF3/Ar/O2. A second layer of EBL was performed (CSAR62) in order to define the device electrodes. Following metalization (3 nm Cr/75 nm Au) via thermal evaporation, a third EBL step was used to open apertures for the deposition of the phase change material. Deposition was carried out using a custom RF sputtering system (Nordiko) where 75 nm GST followed by 5 nm SiO2 were deposited in an Ar environment.

### Measurement Setup

The optical and electrical measurements were conducted using the setup illustrated in Figure 3a. A continuous‐wave (CW) laser source (Keysight N7711A) was used to optically probe the device at 1585 nm. Optical power of 5 mW was coupled into and out of the waveguides through integrated grating couplers with peak transmission at 1585 nm wavelength (see Supporting Information S1). The output of the device was continuously monitored using a 200‐kHz low‐noise photoreceiver (New Focus, Model 2011). A dual‐channel source meter unit (Keithley 2614B) with a constant 100 mV compliance was used in order to simultaneously measure the state of the PCM across the nanogap and measure the current through the nanoheater to ensure no structural changes were incurred. To isolate the electrical readout from the electrical pulses to the device an RF bias tee was used at each measurement channel with the DC port connected to the Keithley and the DC+RF port to the respective device terminal. A 100‐MHz electrical pulse generator was connected to the RF port of the heater bias tee and was controlled by a custom‐built program (LabView). Electrical signals were coupled to the device through RF electrical probes (GGB). Electrical sweeps of multiple devices were conducted with a custom‐built motorized (x,y.z) probe station.

## Conflict of Interest

The authors declare no conflict of interest.

## Author Contributions

N.F. and N.Y. contributed equally to this work. All authors contributed substantially. N.F. performed all fabrication, simulations, and testing of the devices. N.F., N.Y., and H.B. conceived the experiment. A.L. assisted with the electrical measurement of the devices. J.S.L. performed the depositions of the PCM. J.S.L., J.F., S.A., and W.Z. assisted with modeling, characterization, and experimentation. H.B. led the research, with N.F. and H.B. drafting the manuscript, and all authors contributing to the writing of it.

## Supporting information

Supporting Information

## Data Availability

The data that support the findings of this study are available in the supplementary material of this article.
