# Supplementary material for: Electronically Reconfigurable Photonic Switches Incorporating Plasmonic Structures and Phase Change Materials
Source: Adv Sci (Weinh). 2022 Apr 17;9(20):2200383. doi: 10.1002/advs.202200383 (PMC9284156; doi:10.1002/advs.202200383)
Supplement: Supplementary file 1 — Supporting Information [file ADVS-9-2200383-s001.pdf]

Supporting Information

Electronically reconfigurable photonic switches incorporating phase change plasmonics

Nikolaos Farmakidis[1,†], Nathan Youngblood[1,2,†], June Sang Lee[1], Johannes Feldman[1], Alessandro Lodi[1], Xuan Li[1], Samarth Agarwal[1], Wen Zhou[1], Lapo Bogani,[1], Wolfram HP Pernice[3], C David Wright[4] and Harish Bhaskaran[1]*

[1]Department of Materials, University of Oxford, Parks Road, Oxford OX1 3PH, UK.
[2]Present Address: Department of Electrical and Computer Engineering, Swanson School of Engineering, University of Pittsburgh, Pittsburgh, PA 15261, USA
[3]Institute of Physics, University of Munster, Munster, Germany
[4]Departmentof Engineering, University of Exeter, Exeter EX4 4QF, UK.
[†]These authors contributed equally.

*Corresponding author: E-mail: *harish.bhaskaran@materials.ox.ac.uk*

Keywords: Integrated electro-optics, phase change photonics, Mixed-mode PCM

S1. Characteristic IV Behaviour of $Ge_2Sb_2Te_5$ Filled With Increasing Gap Width

S2. Device Transmission Spectra for Amorphous and Crystalline $Ge_2Sb_2Te_5$

S3. Device Optical Micrographs

S4. 3D FDTD Simulation of in Plan View

S1. Characteristic IV Behaviour of $Ge_2Sb_2Te_5$ Filled With Increasing Gap Width

Here the current-voltage characteristics of the GST-filled nanogap are explored. The width of the nanogap is controlled by dose modulation during EBL in order to achieve nanogaps between 50nm and 90nm. For nanogaps which are patterned with a high dose, the gap is closed and displays 3-4 orders of magnitude higher electrical conductivity as compared to nanogaps with 50nm GST. We find that for gaps between 50nm and 90nm the resistance increases with width, spanning the 250 kΩ to 100 MΩ range.

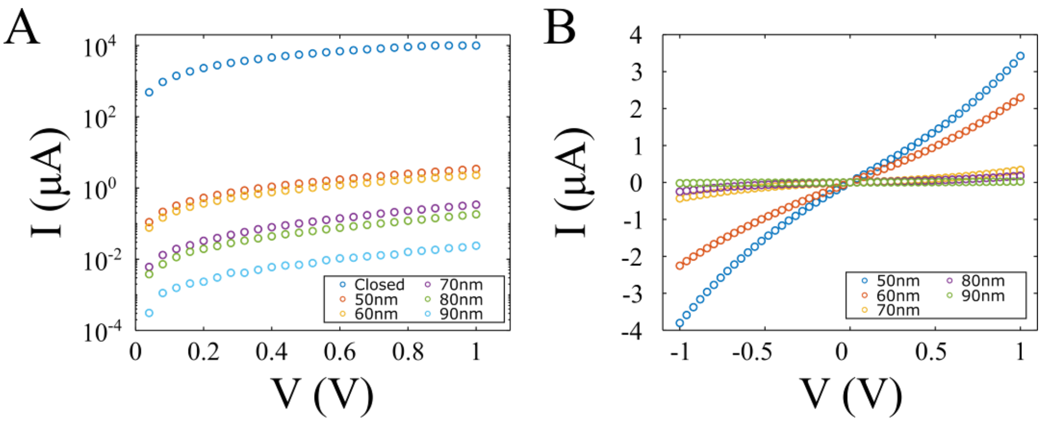

Fig. S1. IV characteristics of devices between 50nm and 90nm in the amorhous state. **(A)** Positive polarity current trends on a semilog scale for six different nanogaps. A closed nanogap displays four orders of magnitude higher conductivity **(B)** The resistance of 50nm-90nm gaps ranges from 250kΩ to 100MΩ.

S2. Device Transmission Spectra for Amorphous and Crystalline $Ge_2Sb_2Te_5$

The optical transmission of fabricated devices is tested as a function of wavelength. Input-output grating couplers are designed for peak transmission at 1590nm such as to achieve high contrast and maintain compatibility with our lasers. As deposited amorphous devices are found to have a transmission of 12% scaled with respect to plain waveguides on chip. Placing the devices on a hotplate for 5minutes at 250°C to induce crystallisation, causes the transmission to be reduced by 4%.

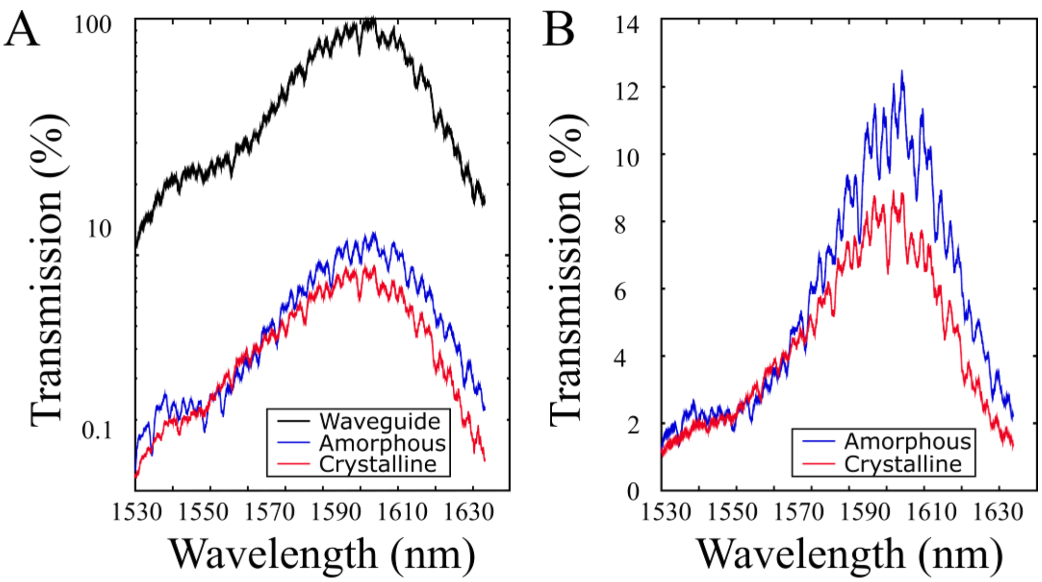

Fig. S2. Optical transmission spectra of a typical device. A) Semi-log plot of the transmission spectra with peak transmission at 1590nm for a plain waveguide used for calibration as well as a device in the amorphous and crystalline states **B)** Linear plot of the transmission spectra. The as deposited device is found to have a transmission of 12% whereas the transmission in the crystalline state reduces to 9%.

S3. Device Optical Micrographs

The general chip outline for the fabricated devices is shown in figure S3 A. For static measurements, the devices were measured before and after switching on a hotplate. In order to account for variabilities in the power coupled to the devices when re-aligning the off-chip optical fibres to the grating couplers on-chip, a plain waveguide located at the boarder of each row of the chip was used for normalisation.

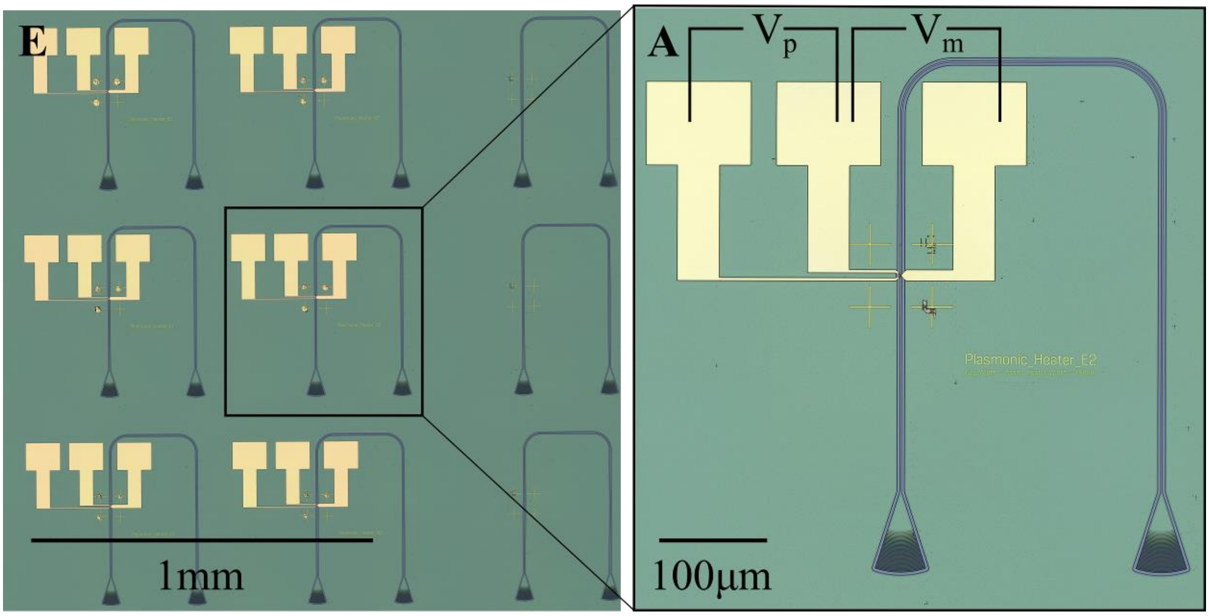

Fig. S3. Optical micrographs of fabricated arrays of devices. (A) Arrays of devices fabricated including a calibration device at the boarder of each row on the chip. **(B)** Optical micrograph of a typical device.

S4. 3D FDTD Simulation of in Plan View

Here we show the electric field distribution of a section along the centre of the nanogap. The field in the nanogap for the case of amorphous GST shows a small attenuation with a loss of 5 dB/µm whereas for the case of crystalline GST the attenuation of the mode increases to 88 dB/µm.

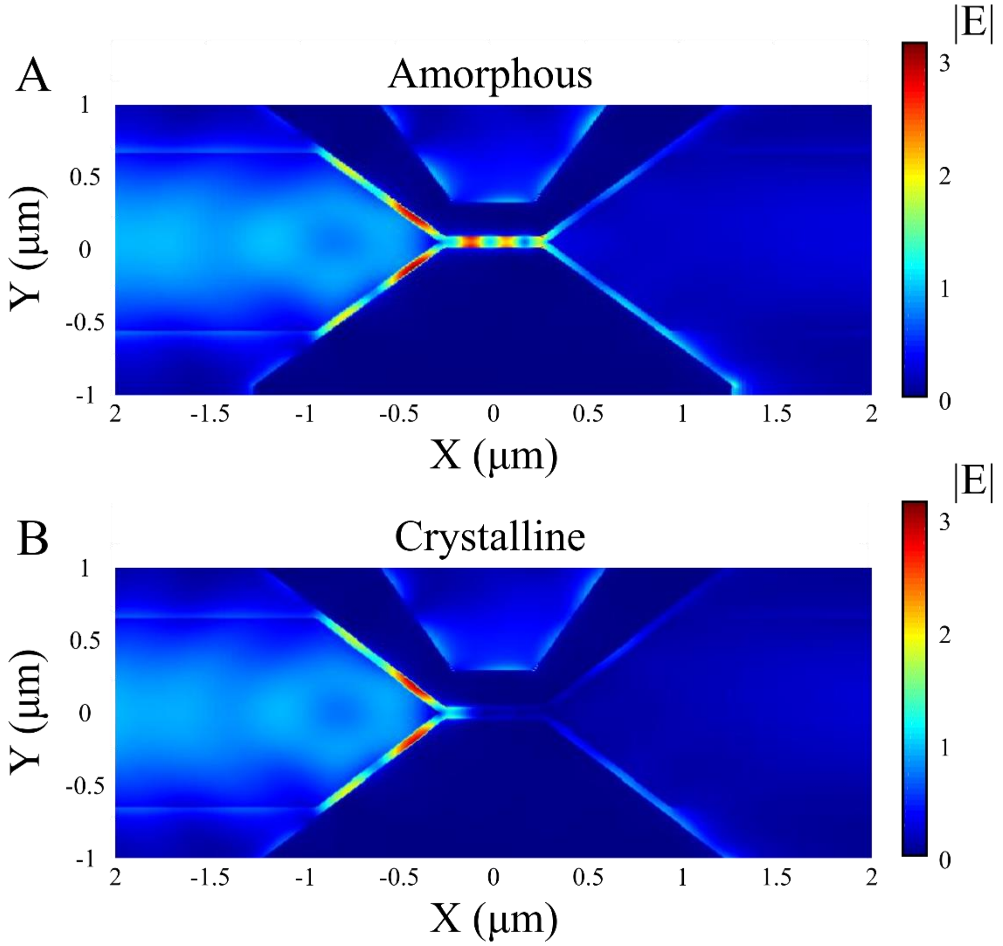

Fig. S4. FDTD simulation of a top-view section of the device. (A) Electric field distribution for amorphous GST **(B)** Electric field distribution for the case of crystalline GST
